# Ultrasound Thickness of Bladder Wall in Continent and Incontinent Women and Its Correlation with Cystometry

**DOI:** 10.1155/2014/684671

**Published:** 2014-11-04

**Authors:** Edney Norio Otsuki, Edward Araujo Júnior, Emerson Oliveira, Marair Gracio Ferreira Sartori, Manoel João Batista Castelo Girão, Zsuzsanna Ilona Katalin Jármy-Di Bella

**Affiliations:** ^1^Department of Gynecology, Escola Paulista de Medicina-Federal University of São Paulo (EPM-UNIFESP), 05303-000 São Paulo, SP, Brazil; ^2^Department of Obstetrics, Escola Paulista de Medicina-Federal University of São Paulo (EPM-UNIFESP), 05303-000 São Paulo, SP, Brazil; ^3^Department of Gynecology and Obstetrics, Faculty of Medicine of ABC, 09060-650 Santo André, SP, Brazil

## Abstract

*Objective*. To compare bladder wall thickness in two kinds of urinary incontinent women—stress urinary incontinence (SUI) and overactive bladder (OAB) with urodynamic detrusor overactivity (DO), and to compare them with continent patients by ultrasound, also, correlate with cystometric results in incontinent women. *Methods*. 91 women were divided into the following groups: continent (*n* = 31), SUI (*n* = 30), and DO (*n* = 30) groups after clinical evaluation and urodynamic test (only in incontinent women). Transvaginal ultrasound was performed to the bladder wall thickness (BWT) measurement. The mean of BWT was calculated and data were analyzed with ANOVA and Turkey's multiple comparison tests. Pearson's correlation coefficient (*r*) was used to compare two variables. Receiver operating characteristic (ROC) curve was performed to study BWT as a diagnostic parameter. *Results*. BWT in DO group was significantly higher than that in the other groups (*P* < 0.005). A moderate positive correlation was found between BWT and maximum bladder pressure during involuntary bladder contraction. There was no difference in BWT between SUI and continent groups. DO group had lower first desire to void and cystometric capacity. Maximum bladder pressure at detrusor contraction had a moderate positive correlation with BWT. The ROC revealed an area under the curve of 0.962 (95% CI, 0.90–1.01). *Conclusions*. DO patients have increased bladder wall thickness, lower first desire to void, and lower cystometric capacity. There was a moderate correlation between BWT and maximum bladder pressure during involuntary bladder contraction.

## 1. Introduction

Urinary incontinence is a common health problem associated with poor perception of personal health, impairment of quality of life, social isolation, and symptoms of depression [1]. The most common subtypes of urinary incontinence are stress urinary incontinence (SUI), with leakage of urine during effort or physical exertion, and urgency urinary incontinence with the complaint of involuntary loss of urine associated with urgency (OAB) [2]. The diagnosis in primary care can be done just based on patient's complaints, or exams may be requested to further investigate the symptoms.

Urodynamic test tries to reproduce the situation in which patients' complaints occur and therefore is considered an extension of patient history and physical examination in a controlled setting; however, it is not a mandatory exam for incontinence diagnosis [3]. Detrusor overactivity (DO) is an urodynamic observation defined by involuntary detrusor contractions during the filling phase, which may be spontaneous or may be provoked. It is detectable in about half of the patients with OAB submitted to urodynamic test [4].

Ultrasound is a diagnostic method that is very much a part of general practice in obstetrics and gynecology and its role in urogynecology has increasing importance [5, 6]. When compared to other imaging exams, it is less invasive, nonradioactive, inexpensive, and widely available. In addition, ultrasound is the gold standard for measuring bladder volume and postvoiding residue, and it allows dynamic assessment of pelvic structures [7, 8].

Bladder wall thickness (BWT) has been studied in incontinent patients and those with OAB especially those with DO who show higher values [9]. Farag and Heesakkers [10], in a literature review, compared the various pathways of ultrasound to measure the BWT and they concluded that the study of BWT by transvaginal transducer is more appropriate. Oelke et al. [11] compared the measurement of BWT obtained by conventional ultrasound with the automatic measurement performed by the BVM 6500 device. Although both show good reproducibility, the conventional measurement showed the smallest variation and it was more reliable. Kuhn et al. [12], comparing different ways to perform ultrasound to measure the BWT, found that vaginal measurement was more reliable than abdominal or perineal assessment.

The objective of this study was to compare the BWT in two kinds of urinary incontinent women, SUI and OAB with DO, and compare them with continent patients by transvaginal ultrasound, also, correlate with cystometric results in incontinent women.

## 2. Material and Methods

In a tertiary referral ambulatory, we selected women who were continent or with SUI or OAB. The study was approved by the Research Ethics Committee of Federal University of São Paulo (UNIFESP), and the volunteer women who agreed to participate gave consent form.

We included continent women with gynecological diseases or conditions other than urinary incontinence like miomas or adnexial cists.

Patients with mixed urinary incontinence, lower urinary tract diseases, bladder outlet obstruction, previous surgery for urinary incontinence, and current or recurrent urinary tract infection were not included. All patients were examined and pelvic organ prolapse quantification (POP-Q) was determined. Urodynamic test was performed before inclusion in this study and it followed the International Continence Society (ICS) recommendations using Dynapack MPX 816 four-channel urodynamic system (Dynamed, Sao Paulo, Brazil). The clinical diagnosis had to match the urodynamic test result.

**Figure 1:**
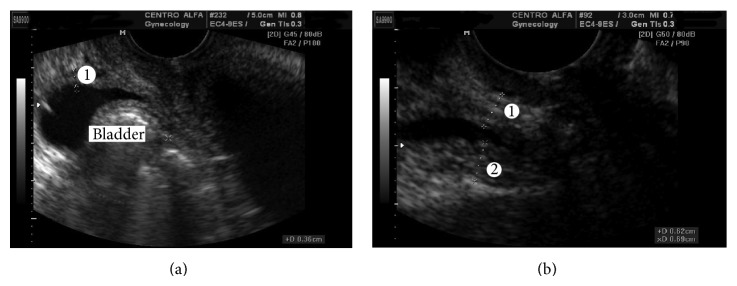
Two-dimensional transvaginal ultrasound. (a) Measurement of anterior wall thickness of bladder (1). (b) Measurement of thickness wall in trigone bladder (1); measurement of thickness wall in dome bladder.

Women were asked to void. Then, in supine position, ultrasound exam was performed using a SA-9900 (Samsung, Seoul, Korea) ultrasound machine equipped with a multifrequential transvaginal transducer (4–9 MHz). Using the method described by Haylen et al. [13], the residual volume was calculated to ensure that it was <50 mL. The bladder was visualized in the sagittal plane and then the probe was laterally moved 1 cm to achieve a clear view of the bladder and directed cranially to image the bladder in the parasagittal plane. At maximal magnification, the measurements were made perpendicular to the luminal surface of the bladder in the thickest part of trigone, dome of the bladder, and anterior wall of the bladder (Figure 1). BWT was considered the mean value of these three measurements. All exams were performed by the same physician (ENO, urogynecologist) who was also blinded to the incontinence diagnosis.

The data were transferred to the spreadsheet program Excel 2007 (Microsoft Corp., Redmond, WA, USA) and the statistical analysis was performed with GraphPad Prism version 5.0 for Windows (GraphPad Software, San Diego, CA). Analysis of variance (ANOVA) or Student's *t*-test was used to compare continuous variables. Post hoc multiple comparisons were performed using the Tukey's multiple comparison tests. Pearson's chi-squared test (*χ* ^2^) was used to compare categorical variables. Mann-Whitney *U* test was applied to compare two independent random samples. Pearson's correlation coefficient (*r*) was used to compare linear dependence between two variables. In all analyses, we used a significance level of *P* < 0.05.

## 3. Results

A total of 91 patients with age between 18 and 81 were included in this study. Of these, 31 were continent, 30 had SUI, and 30 had urgency urinary incontinence (OAB with DO).

Clinical characteristics of the groups are listed in Table 1. There was no difference in age (*P* = 0.258), hormonal status (*P* = 0.412), body mass index (*P* = 0.474), parity (*P* = 0.492), or POP-Q (*P* = 0.738) among the groups. Postvoid residual ranged from 0 to 40 mL.

**Figure 2:**
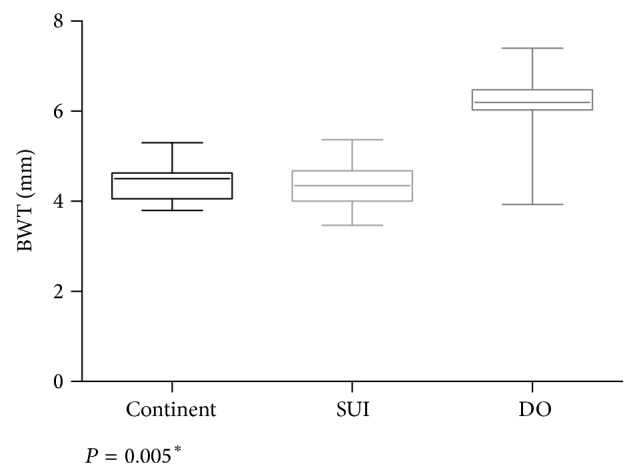
Boxplot of the bladder wall thickness (BWT) according to the groups. ^*^SUI: stress urinary incontinence; DO: detrusor overactivity.

BWT was higher in detrusor overactivity group and significantly different compared to SUI and continent groups (*P* = 0.005). There was no difference between SUI and continent groups (Figure 2).

In Table 2, urodynamic findings are listed. In SUI group, 3 patients had intrinsic sphincter deficiency. In DO group, first desire to void and maximum cystometric capacity were significantly lower compared to SUI results.

The linear dependence in SUI group between BWT and first desire to void (*r* = 0.16, *P* = 0.216), volume at leakage (*r* = −0.10, *P* = 0.304), and maximum cystometric capacity (*r* = 0.01, *P* = 0.462) was not significant.

**Figure 3:**
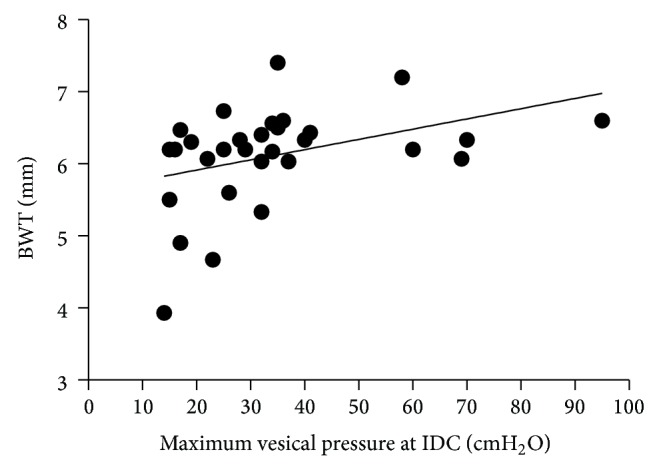
Scatter plot of correlation between bladder wall thickness (BWT) and maximum vesical pressure at involuntary detrusor contraction (IDC).

Concerning OAB group, there was a moderate positive correlation between BWT and maximum vesical pressure at involuntary detrusor contraction (*r* = 0.39, *P* = 0.017) (Figure 3). There was no significant correlation between BWT and first desire to void (*r* = 0.14, *P* = 0.242) and maximum cystometric capacity (*r* = −0.10, *P* = 0.308).

## 4. Discussion

The diagnosis of the type of incontinence only with the clinical assessment of patients can mislead to the right treatment. It is not uncommon that patients with OAB have involuntary detrusor contractions triggered by stress maneuver such as coughing or sneezing. In these cases, particularly, Giarenis et al. [14] observed worse efficacy with tolterodine than women with involuntary detrusor contractions during cystometric filling phase of urodynamic test. It takes a good amount of self-perception to tell if the urinary loss is caused by sudden increase in intraabdominal pressure or by a detrusor contraction. Also, different diseases share common lower urinary tract symptoms and many times a consistent diagnosis is not reached [3, 15, 16]. Questionnaires of quality of life, bladder diaries, and visual analog scales are valuable to expand the understanding and bothersome of the symptoms but provide only subjective data.

Currently, when clinical assessment of urinary incontinence is compared to urodynamic test, there is lack of a gold standard [17]. Clarke [18] considered the urodynamic test inappropriate to diagnose and introduced treatment based only in symptoms, as he found DO in 64% of the patients with SUI. Urodynamic test can provide valuable information of the underlying pathophysiology, but its intra- and interobserver reproducibility is not good [19, 20].

Healthy patients have BWT measurement ranging from 3 to 5 mm [21]. Conditions such as infection, pelvic radiation, pelvic surgery, neurological disease, cancer, and bladder outlet obstruction can cause thickening of the bladder wall associated to other sonographic signals [22]. The study of BWT has brought the question if it is possible to diagnose OAB by a “cut-off” value measured by ultrasound. Khullar and Cardozo [23] proposed 5 mm as a cut-off value to discriminate detrusor overactivity, while Robinson et al. [5] considered 6 mm to the diagnose of OAB in patients without evidence of SUI. Kuhn et al. [24] with a cut-off value of 5.6 mm found sensitivity of 83.3% and specificity of 87.5% in distinguishing between OAB and bladder obstruction. Serati et al. [25] compared BWT in different forms of incontinence and concluded that a “cut-off” value of 6.5 mm could distinguish patients with detrusor overactivity, pure or associated with SUI, but it could not replace urodynamic test.

An important question is if we are looking to the right point. The diagnosis of DO is controversy. DO is present in only 40–60% of the OAB patients [26]. Although urodynamic test is the only method to its diagnosis, the interobserver variation in evaluation of the same exam is high [27]. DO is not exclusive of OAB patients; asymptomatic patients may present DO during urodynamic test when the saline solution is instilled very fast or too cold.

It is unknown if involuntary detrusor contraction is the cause of urgency at storage phase or that other types of lower urinary tract dysfunction can cause OAB complaints [28]. Normal bladder physiology includes phasic contractions of low magnitude with frequency of dozens per minute, thus promoting a better adjustment of the bladder surface to urine filling [29]. The detrusor of OAB patients presents biochemical changes, which have different cellular ultrastructure and tissue macrostructure leading to more contractility and resulting in hypertrophy of the detrusor muscle [30].

Using ICS's definition of OAB syndrome, based only on symptoms, can lead to a heterogeneous group, as women with different physiopathologies present with the core complaint of urinary urgency. In a different approach, we aimed to study a homogeneous group only including women who not only complained of urgency, but were also incontinent and with presence of DO at urodynamic test. Women with DO experience more significant impairment to their quality of life and have a greater degree of bladder dysfunction [31]. Our finding of higher values of BWT associated with higher vesical pressure at involuntary detrusor contraction concurs with the background of OAB. Also, there was a moderate positive relation between intensity of OAB and BWT.

We decided not to include patients with mixed urinary incontinence because of the difficulty to determine the “amount” of troublesome of each component of this group.

The time lapse necessary to increase the BWT from normal values is a question that remains without response. To our knowledge, it is not demonstrated yet.

According to the definition of OAB suggested by the ICS, the need to detect DO loses importance. On the other hand, it seems that increased BWT, as a result of repeated DO, is linked to clinical symptoms, as Panayi et al. [32] observed that women with BWT greater than 5 mm had a visual analog scale of urgency significantly higher when compared to controls. In a recent article, Abou-Gamrah et al. [33] found 4.78 mm of BWT the best cut-off value for prediction of OAB in patients with lower urinary tract symptoms.

As a diagnostic tool of urinary incontinence, we agree that BWT cannot replace urodynamic test. Nonetheless, our findings encouraged us to consider it as an “index” of detrusor activity.

One of the strengths of this paper is the observation that the BWT of the SUI patients and that of normal women are similar and significantly thinner than that of the DO patients, the most severe form of OAB. For our knowledge this is the first study that observed the BWT in SUI. The urodynamic evaluation in incontinent women and correlation with ultrasound findings are also the strengths of this paper. On the other hand, the weakness of this paper was that we did not investigate BWT of mixed urinary incontinence patients.

In summary, we believe that the thickening of the bladder wall is itself an important hallmark in OAB patients mainly when the clinic is not compatible with urodynamic test or in women not responsive to anticholinergics.

## Figures and Tables

**Table 1:** Clinical characteristics of the patients.

Variable	*N* = 91
Age	50.5 ± 14.40
Menopause	51 (56)
BMI	27.03 ± 5.63
Parity	3.29 ± 2.88

Data presented as mean ± SD; data in parenthesis are percentages. BMI: body mass index.

**Table 2:** Urodynamic findings in women with stress urinary incontinence (SUI) or overactive bladder (OAB) with detrusor overactivity (DO).

Variable	SUI (*n* = 31)	DO (*n* = 30)	*P* value^*^
Volume at first desire to void (mL)	157.7 ± 60.8	124.4 ± 56.27	0.002^*^
Maximum cystometric capacity (mL)	471.6 ± 100.5	339.2 ± 135.6	<0.001^*^
Valsalva leak point pressure (cmH_2_O)	87.7 ± 28.93		
Volume at leakage (mL)	301.8 ± 164.7		
Cystometric volume at first IDC (mL)		242.2 ± 117.1	
Maximum vesical pressure at IDC (cmH_2_O)		34.96 ± 19.8	

Data presented as mean ± SD; data in parenthesis are percentages. IDC: involuntary detrusor contraction. ^*^Student's *t*-test.
